# Network analysis of anxiety and depression in the functionally impaired elderly

**DOI:** 10.3389/fpubh.2022.1067646

**Published:** 2022-12-01

**Authors:** Tianqi Yang, Zhihua Guo, Xiaoqin Cao, Xia Zhu, Qin Zhou, Xinhong Li, Hui Wang, Xiuchao Wang, Lin Wu, Shengjun Wu, Xufeng Liu

**Affiliations:** ^1^Department of Military Medical Psychology, Air Force Medical University, Shaanxi, China; ^2^Xijing Hospital, Air Force Medical University, Shaanxi, China; ^3^Tangdu Hospital, Air Force Medical University, Shaanxi, China

**Keywords:** anxiety, depression, elderly, network analysis, functional impairment

## Abstract

**Background:**

Evidence from previous studies has confirmed that functionally impaired elderly individuals are susceptible to comorbid anxiety and depression. Network theory holds that the comorbidity emerges from interactions between anxiety and depression symptoms. This study aimed to investigate the fine-grained relationships among anxiety and depression symptoms in the functionally impaired elderly and identify central and bridge symptoms to provide potential targets for intervention of these two comorbid disorders.

**Methods:**

A total of 325 functionally impaired elderly individuals from five communities in Xi'an, China, were recruited for our investigation. The GAD-7 and PHQ-9 were used to measure anxiety and depression, respectively. SPSS 22.0 software was used for descriptive statistics, and R 4.1.1 software was used for network model construction, expected influence (EI) evaluation and bridge expected influence (BEI) evaluation.

**Results:**

In the network, there were 35 edges (indicating partial correlations between symptoms) across the communities of anxiety and depression, among which the strongest edge was A1 “Nervousness or anxiety”-D2 “Depressed or sad mood.” A2 “Uncontrollable worry” and D2 “Depressed or sad mood” had the highest EI values in the network, while A6 “Irritable” and D7 “Concentration difficulties” had the highest BEI values of their respective community. In the flow network, the strongest direct edge of D9 “Thoughts of death” was with D6 “Feeling of worthlessness.”

**Conclusion:**

Complex fine-grained relationships exist between anxiety and depression in functionally impaired elderly individuals. “Uncontrollable worry,” “depressed or sad mood,” “irritable” and “concentration difficulties” are identified as the potential targets for intervention of anxiety and depression. Our study emphasizes the necessity of suicide prevention for functionally impaired elderly individuals, and the symptom “feeling of worthlessness” can be used as an effective target.

## Introduction

Functionally impaired elderly individuals are those who cannot independently perform any activities of daily living, such as bathing, transfers from bed and chair, toileting, dressing, feeding, etc., due to their old age, weakness, disability, illness, mental retardation, etc. (1, 2). According to a report issued by the China Aging Scientific Research Center and Social Sciences Academic Press (3), there are more than 40 million functionally impaired elders in China as of 2015, accounting for 18.3% of the elderly population. The aging world population has brought escalating social and economic burdens (4). Functionally impaired elders not only fail to undertake basic personal care tasks but are also susceptible to psychiatric comorbidities, such as anxiety and depression (5–10).

Anxiety and depression are closely associated with a range of negative consequences and possible dangers such as Parkinson's disease and cardiovascular disease (11, 12). Furthermore, anxiety and depression contribute to the increasing likelihood of suicidal ideation and suicide attempts for both adolescents (13) and functionally impaired elders (14, 15). The high prevalence and undesirable consequences of these two disorders make them a large global burden of disease (16), contributing substantially to the increasing health care costs and low quality of life for the functionally impaired elderly. Therefore, anxiety and depression in the functionally impaired elderly population warrant more attention. For clinical applications, efforts are needed to determine effective intervention and treatment methods for the two psychiatric disorders.

It is widely known that anxiety and depression are frequently comorbid disorders (17–20), with co-occurrence rates ranging from 10 to 90% (21, 22). Patients with comorbid anxiety and depression are often more severely ill than patients with only one disorder. This population does not respond well to treatment, has a longer duration of illness, and experiences poor prognosis (22). It has been revealed that anxiety and depression trigger each other and are bidirectional risk factors for one another (23, 24). Therefore, simply targeting only one disorder may not be effective because anxiety and depression are mutually enhanced and facilitated, like coinfections (25), making it difficult to treat them separately. Additionally, the majority of previous studies focus on anxiety and depression based on sum-scores, masking the heterogeneity between different symptoms and obscuring the symptom-level relationships (26–28). Analysis of individual symptoms and their interactions provides a promising way forward. In summary, high comorbidity of anxiety and depression emphasizes the need for identifying underlying mechanisms of these two co-occurring disorders and targeted symptoms at a fine-grained level for more effective treatment, for example, the symptoms critical to linking anxiety and depression or symptoms playing important roles in developing both anxiety and depression.

Network analysis, an emerging data-driven approach to psychopathology and comorbidity, is ideally suited for the purpose of this study (29–31). In the network model, psychiatric disorders are constructed as networks emerging from interactions between symptoms, which means that the symptoms and their interactions actively contribute to the development and maintenance of disorders rather than passively reflecting the latent variable (30, 31). The network is composed of two basic elements, namely, the nodes (symptoms) and the node-to-node edges (associations between symptoms) (32). It helps to investigate the characteristics of comorbid systems and the fine-grained relationships between individual symptoms from the perspective of mathematics and visualize them intuitively (33–35). Network analysis offers important new perspectives on comorbidity (36). This approach involves assessing the important edges that may shed light on the psychiatric pathways between comorbidities *via* edge weights. The approach also makes it possible to identify central nodes that activate all other symptom nodes, exert great influence on the overall network and determine bridge symptoms that play significant roles in maintaining the co-occurrence of mental disorders and facilitating the spread of comorbidity; these identified symptoms represent promising and effective targets for intervention and treatment (35, 37–39).

To date, several studies have examined the network structure of anxiety and depression in a joint framework in different populations, including patients with major depressive disorder (40), patients with anxiety disorders (41, 42), epilepsy patients (43, 44), and nursing students (45, 46). However, the anxiety and depression symptom interactions of functionally impaired elderly individuals have not been researched *via* network analysis. Considering the different self-reported scales used, various study populations, and the data-driven nature of the network methodology, the findings are specific to the samples included in these studies and can hardly be generalized to functionally impaired elderly individuals. Consequently, studies are necessary to examine the relationships between anxiety and depression in functionally impaired elderly individuals and determine promising targets for intervention and treatment.

To address this research gap, the current study is the first to construct a symptom-level structure of anxiety and depression in functionally impaired elderly individuals using network analysis. Herein, we wanted to examine the strongest edges between anxiety and depression symptoms, the most influential nodes that maintain the whole anxiety-depression network, and the critical nodes that bridge anxiety and depression communities. The purpose of the study is to advance our understanding of the fine-grained relationships between anxiety and depression and to determine effective therapeutic targets for these two comorbid disorders.

## Materials and methods

### Participants

From March to August 2022, a total of 325 functionally impaired elderly individuals from 5 communities in Xi'an, China, were recruited by the convenience sampling method. The inclusion criteria were: age 60 years or older, Barthel Index score <100, elderly care at home for more than 6 months, clear awareness, barrier-free communication, and informed and voluntary participation in the survey. Those who could not cooperate with the investigation, such as functionally impaired elderly with severe mental illness, cognitive dysfunction and severe physical disease, were excluded. The systematically trained investigators conducted face-to-face household questionnaires in the 5 communities which have continuous nursing cooperation with us. The Generalized Anxiety Disorder-7 (GAD-7) and the Patient Health Questionnaire-9 (PHQ-9) were used to investigate the functionally impaired elderly after making them clear about the purpose and method of the study by using unified guidelines. A total of 335 questionnaires were distributed, and 325 valid questionnaires were collected with an effective recovery rate of 97.01%. The current study followed the Helsinki Declaration and was approved by the Ethics Committee of Xijing Hospital, Air Force Medical University (No. KY20222194-C-1).

### Measurements

#### Generalized anxiety disorder-7 (GAD-7)

According to DSM-IV (47), GAD-7 (48) is used to assess the most important diagnostic symptoms of anxiety within the last 2 weeks. It contains 7 items, such as “Feeling nervous, anxious or on edge.” The items are rated on a 4-point Likert-type scale with “not at all,” “on some days,” “on more than half of the days” and “almost every day” scored 0–3, respectively. Higher scores on the GAD-7 indicate more severe symptoms of anxiety. In the present study, the Cronbach's α coefficient of the GAD-7 was 0.87.

#### Patient health questionnaire-9 (PHQ-9)

The PHQ-9 (49) is a widely used evaluation tool for depression symptoms recommended by DSM-IV (47). It consists of nine items, such as “Thoughts that you would be better off dead or of hurting yourself in some way.” The items are rated on a 4-point Likert-type scale with “not at all,” “on some days,” “on more than half of the days” and “almost every day” scored 0–3, respectively. Higher scores on the PHQ-9 suggest more severe symptoms of depression. The Cronbach's α coefficient of the PHQ-9 in the present study was 0.84.

### Data analysis

SPSS 22.0 software was used to calculate the means, standard deviations and Cronbach's α coefficients on the GAD-7 and PHQ-9. R 4.1.1 software was used for network model construction, expected influence (EI) evaluation and bridge expected influence (BEI) evaluation.

#### Network model construction

We used the R package qgraph (50) to construct a Gaussian graphical model (GGM) of the anxiety-depression network in functionally impaired elderly individuals (32). Least absolute shrinkage and selection operator (LASSO) (51) regularization and the extended Bayesian information criterion (EBIC) (52) were applied to shrink all edges and set small spurious edges exactly to zero (32). The tuning parameter of the EBIC keeps the balance between including false edges and removing true edges (33), and it was set to 0.5 according to the recommendation of Foygel and Drton (53). The Spearman rho correlation method was used in the network construction. The nodes in the network represented items of GAD-7 and PHQ-9 and were divided into two communities according to their theoretical sources, namely, the anxiety community and the depression community. The correlations of symptoms were represented by edges, and the calculation of the correlation between two nodes was conducted after statistical control for the influence of all the other nodes included in the network (54). Blue edges and red edges represent positive and negative correlations, respectively, and thicker edges represent higher correlations. The Fruchterman-Reingold algorithm was used to arrange the network layout; in this layout, strong correlations are placed in the center of the network, and weak correlations are placed in the periphery of the network (55). The graphics function “flow” of the R package qgraph was used to display clearly which symptoms of anxiety and depression are directly or indirectly correlated with the depression symptom “thoughts of death.”

We used the R package bootnet (32) to test the significance of the difference in edge weights of different node pairs and estimate the accuracy of edge weights. The difference in edge weights of different node pairs was tested by using bootstrapping (1,000 bootstrapped samples, α = 0.05). The accuracy of edge weights was evaluated by estimating the 95% confidence interval using nonparametric bootstrapping (1,000 bootstrapped samples). According to Epskamp et al. (32), a good accuracy of the edge weights is represented by a narrow confidence interval.

#### EI evaluation

We used the R package qgraph (50) to evaluate the EIs of nodes in the anxiety-depression network of the functionally impaired elderly. As one of the centrality indices, the EI of a specific node is defined as the sum of all the edges that extend from this given node (56). EI reflects the importance of the given node in the network (46) and was selected in our study because it is particularly suitable for networks containing positive and negative edges (30).

We used the R package bootnet (32) to test the stability of EI and the significance of difference on EIs of nodes. The stability of EI was tested by using case-dropping bootstrapping (1,000 bootstrapped samples) and quantitatively estimated by the correlation stability (CS) coefficient. A CS coefficient >0.5 indicates that the stability is ideal (32). The difference in EIs of different nodes was tested by using bootstrapping (1,000 bootstrapped samples, α = 0.05).

#### BEI evaluation

We used the R package networktools (35) to evaluate the BEIs of nodes in the anxiety-depression network of the functionally impaired elderly. BEI is defined as the sum of the cross-community edge weights of the given node. A higher value of BEI indicates stronger connections with nodes of the other community (57).

We used the R package bootnet (32) to test the stability of BEI and the significance of difference on BEIs of nodes. The stability of the BEI was tested by using case-dropping bootstrapping (1,000 bootstrapped samples) and quantitatively estimated by the correlation stability (CS) coefficient. A CS coefficient >0.5 indicates that the stability is ideal (32). The difference in BEIs of different nodes was tested by using bootstrapping (1,000 bootstrapped samples, α = 0.05).

## Results

### Descriptive statistics

The demographic characteristics of the participants are shown in Table 1. The means, standard deviations, EIs and BEIs of the symptoms in the anxiety-depression network of the functionally impaired elderly are shown in Table 2.

**Table 1:** Demographic characteristics of the functionally impaired elderly (*n* = 325).

**Variables**	**Mean (SD), range, %**
Age	75.03 (9.74), 60–97
**Gender**	
Male	180, 55.38%
Female	145, 44.62%
**Educational background**	
Education without schooling	37, 11.38%
Primary school education	54, 16.62%
Junior high school education	91, 28.00%
High school education	73, 22.46%
University education	70, 21.54%
**Marital status**	
Unmarried	2, 0.62%
Married	250, 76.92%
Divorced	5, 1.54%
Widowed	68, 20.92%

**Table 2:** The means, standard deviations, expected influences and bridge expected influences of the symptoms in the anxiety-depression network.

**Symptoms**	**M**	**SD**	**EI**	**BEI**
**Anxiety symptoms**	
A1: Nervousness or anxiety	1.06	0.90	0.88	0.35
A2: Uncontrollable worry	0.61	0.81	1.04	0.18
A3: Worry too much	0.46	0.73	0.81	0.17
A4: Trouble relaxing	0.52	0.75	0.75	0.07
A5: Restlessness	0.44	0.70	0.99	0.27
A6: Irritable	0.54	0.76	0.87	0.41
A7: Afraid something will happen	0.59	0.80	0.95	0.29
**Depression symptoms**	
D1: Anhedonia	1.30	0.90	0.95	0.07
D2: Depressed or sad mood	1.28	0.92	1.30	0.31
D3: Sleep difficulties	1.26	1.04	0.54	0.21
D4: Fatigue	0.97	0.84	0.87	0.10
D5: Appetite changes	0.68	0.81	0.51	0.12
D6: Feeling of worthlessness	0.86	0.88	0.94	0.24
D7: Concentration difficulties	0.61	0.80	1.00	0.48
D8: Psychomotor agitation/retardation	0.56	0.80	0.56	0.04
D9: Thoughts of death	0.42	0.71	0.79	0.17

The maximum possible value on the items is 3.

### Network analysis

#### Structure of the anxiety-depression network

**Figure 1:**
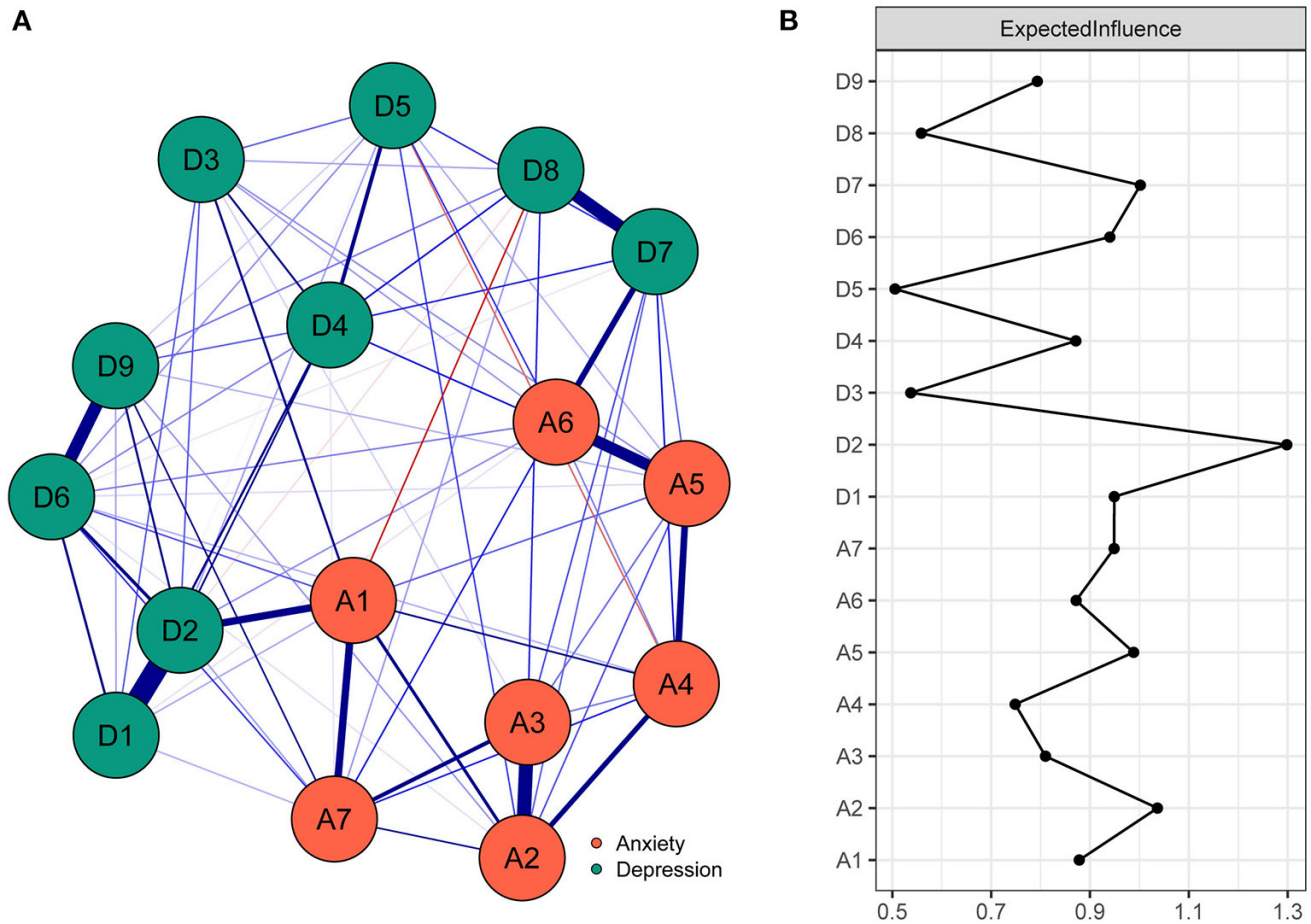
The anxiety-depression network structure and the expected influence indices in the functionally impaired elderly. **(A)** The anxiety-depression network structure of the functionally impaired elderly. The nodes in the network represent symptoms of anxiety and depression, and the edges represent correlations of symptoms. Blue edges and red edges represent positive and negative correlations, respectively, and thicker edges represent higher correlations. **(B)** The expected influence indices in the anxiety-depression network of the functionally impaired elderly (raw score). A1, Nervousness or anxiety; A2, Uncontrollable worry; A3, Worry too much; A4, Trouble relaxing; A5, Restlessness; A6, Irritable; A7, Afraid something will happen; D1, Anhedonia; D2, Depressed or sad mood; D3, Sleep diculties; D4, Fatigue; D5, Appetite changes; D6, Feeling worthlessness; D7, Concentration diculties; D8, Psychomotor agitation/retardation; D9, Thoughts of death.

The anxiety-depression network of functionally impaired elderly individuals is displayed in Figure 1A. Theoretically, there are up to 120 possible edges in this network, while our study found only 78 non-zero edges (edge weight ranging from −0.09 to 0.55). Among these edges, 35 edges (44.87%) were across communities, and 43 edges (55.13%) were within communities. Most of the cross-community edges were positive, of which the 10 edges with the highest edge weight were A1 “Nervousness or anxiety”-D2 “Depressed or sad mood” (edge weight = 0.23), A6 “Irritable”-D7 “Concentration difficulties” (edge weight = 0.20), A1 “Nervousness or anxiety”-D3 “Sleep difficulties” (edge weight = 0.11), A4 “Trouble relaxing”-D7 “Concentration difficulties” (edge weight = 0.10), A7 “Afraid something will happen”-D9 “Thoughts of death” (edge weight = 0.10), A3 “Worry too much”-D8 “Psychomotor agitation/retardation” (edge weight = 0.09), A5 “Restlessness”-D4 “Fatigue” (edge weight = 0.09), A6 “Irritable”-D5 “Appetite changes” (edge weight = 0.08), A7 “Afraid something will happen”-D6 “Feeling of worthlessness” (edge weight = 0.08) and A2 “Uncontrollable worry”-D5 “Appetite changes” (edge weight = 0.07). It is worth mentioning that there were 2 negative cross-community edges, namely, A1 “Nervousness or anxiety”-D8 “Psychomotor agitation/retardation” (edge weight = −0.09) and A4 “Trouble relaxing”-D5 “Appetite changes” (edge weight = −0.06). Supplementary Table 1 in the Supplementary material shows more detailed information on the network structure.

As shown in Supplementary Figure 1 in the Supplementary material, the accurate estimation of edge weights was proven by the narrow bootstrapped 95% confidence interval. The results of the bootstrapped difference test for edge weights are shown in Supplementary Figure 2 in the Supplementary material.

#### The central symptoms

As shown in Figure 1B, A2 “Uncontrollable worry” and D2 “Depressed or sad mood” had the highest EI values (1.04, 1.30) in the anxiety-depression network of the functionally impaired elderly and were identified as central symptoms. In addition, the EIs of A2 “Uncontrollable worry” and D2 “Depressed or sad mood” were significantly larger than those of 33 to 100% of all the other symptoms in the network (see Supplementary Figure 3 in the Supplementary material, *P* < 0.05). The CS coefficient of EI was 0.60, which indicated ideal stability of EI estimation (see Supplementary Figure 4 in the Supplementary material).

#### The bridge symptoms

**Figure 2:**
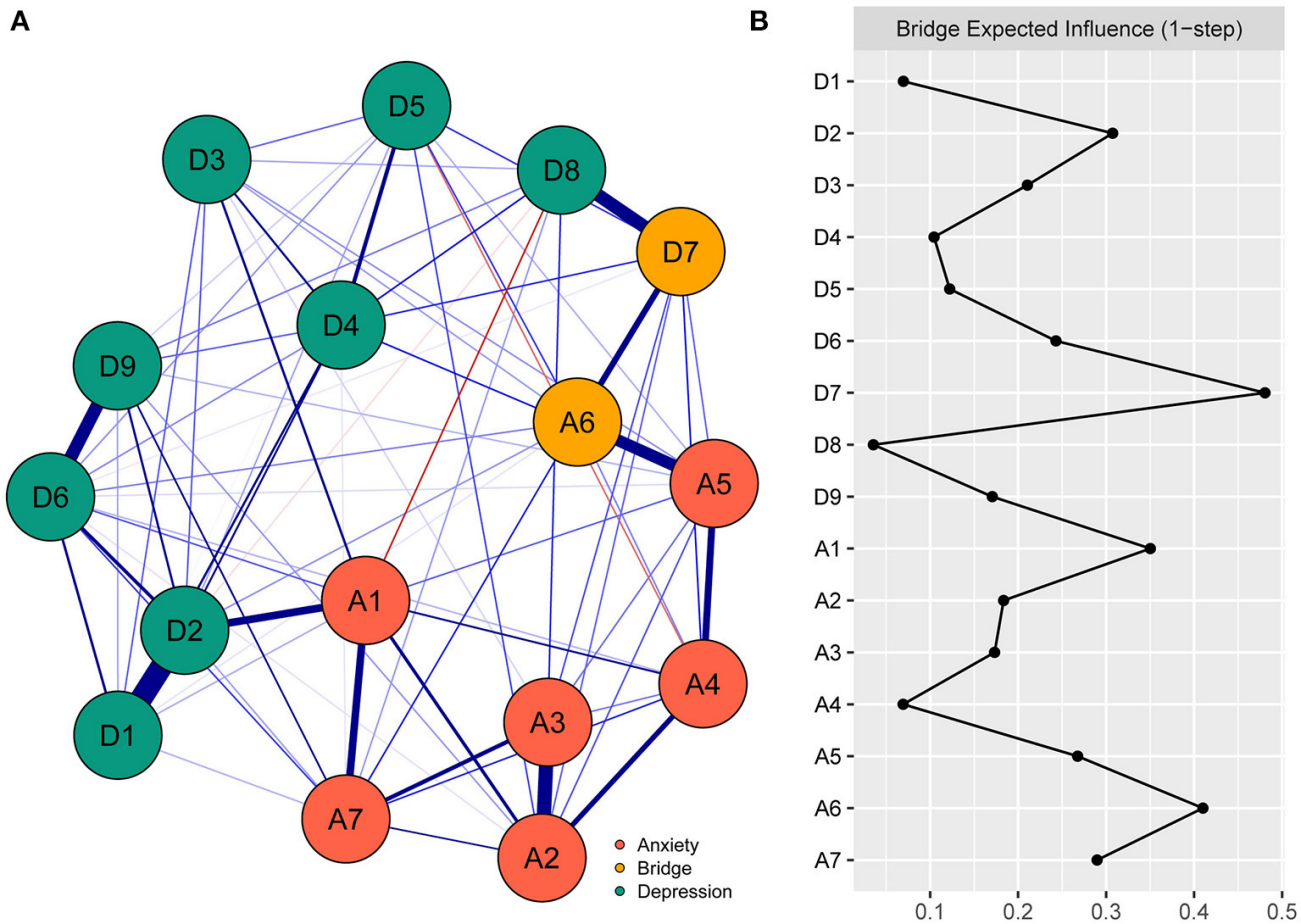
The anxiety-depression network structure highlighting the bridge symptoms and the bridge expected influence indices in the functionally impaired elderly. **(A)** The anxiety-depression network structure highlighting the bridge symptoms. The nodes in the network represent symptoms of anxiety and depression, and bridge symptoms are highlighted in orange. The edges represent correlations of symptoms, blue edges and red edges represent positive and negative correlations, respectively, and thicker edges represent higher correlations. **(B)** The bridge expected influence indices in the anxiety-depression network of the functionally impaired elderly (raw score). A1, Nervousness or anxiety; A2, Uncontrollable worry; A3, Worry too much; A4, Trouble relaxing; A5, Restlessness; A6, Irritable; A7, Afraid something will happen; D1, Anhedonia; D2, Depressed or sad mood; D3, Sleep diculties; D4, Fatigue; D5, Appetite changes; D6, Feeling worthlessness; D7, Concentration diculties; D8, Psychomotor agitation/retardation; D9, Thoughts of death.

The bridge symptoms of the anxiety-depression network of functionally impaired elderly individuals are shown in Figure 2A. As shown in Figure 2B, A6 “Irritable” (0.41) and D7 “Concentration difficulties” (0.48) were the symptoms with the highest BEIs in their own community and were identified as bridge symptoms. In addition, the BEIs of A1 “Nervousness or anxiety”, A6 “Irritable” and D7 “Concentration difficulties” were significantly larger than those of 33% to 73% of all the other symptoms in the network (see Supplementary Figure 5 in the Supplementary material, *P* < 0.05). The CS coefficient of BEI was 0.52, which indicated ideal stability of BEI estimation (see Supplementary Figure 6 in the Supplementary material).

#### Flow network of death thoughts

**Figure 3:**
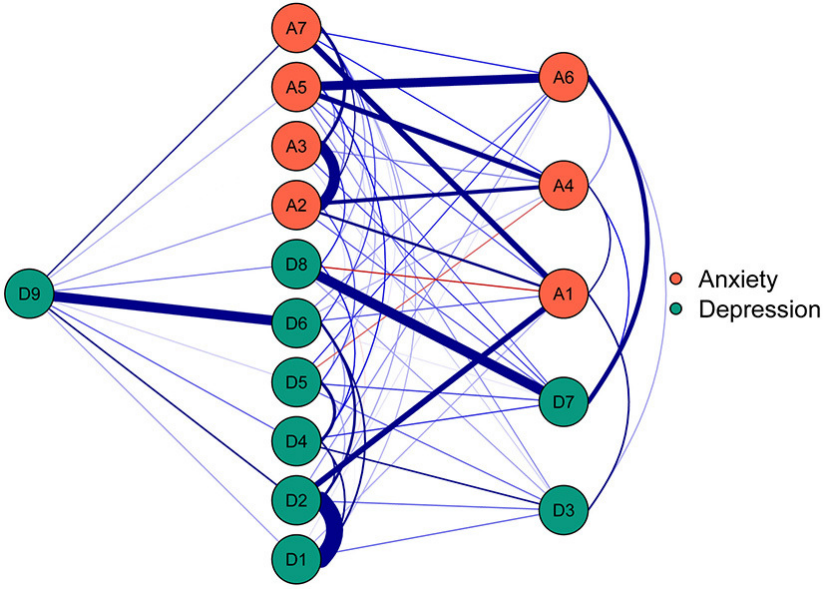
The flow network of death thoughts in the functionally impaired elderly. The nodes in the network represent symptoms of anxiety and depression, and the edges represent correlations of symptoms. Blue edges and red edges represent positive and negative correlations, respectively, and thicker edges represent higher correlations.

The flow network of D9 “Thoughts of death” is shown in Figure 3. There were 10 symptoms directly related to D9 “Thoughts of death” and 5 symptoms indirectly related to D9 “Thoughts of death.” Among the direct edges with D9 “Thoughts of death”, the strongest edges were with D6 “Feeling of worthlessness” (edge weight = 0.35), D2 “Depressed or sad mood” (edge weight = 0.11) and A7 “Afraid something will happen” (edge weight = 0.10).

## Discussion

In the current study, we built a network model to explore the fine-grained relationships between anxiety and depression in functionally impaired elderly individuals. We estimated the EI and BEI indices to find the potential intervention targets for anxiety and depression. We constructed a flow network to intuitively display the anxiety and depression symptoms directly or indirectly related to death thoughts to provide reliable suggestions for suicide prevention. Considering that no studies have investigated the relationships between anxiety and depression symptoms among functionally impaired elderly individuals, our study is largely exploratory and only provides initial insights into this question.

### The fine-grained relationships between anxiety and depression

The cross-community edges displayed in the network structure represented the fine-grained relationships between anxiety and depression, and these relationships help maintain comorbidity and may reflect the potential interactions of anxiety and depression in the functionally impaired elderly (58, 59). Therefore, the crucial edges connecting the anxiety and depression communities are discussed.

In the present study, A1 “Nervousness or anxiety” was positively correlated with D2 “Depressed or sad mood” and D3 “Sleep difficulties” and negatively correlated with D8 “Psychomotor agitation/retardation.” The correlation between “nervousness or anxiety” and “depressed or sad mood” has been confirmed by previous studies (33). As the core diagnostic symptoms of anxiety and depression, respectively, “Nervousness or anxiety” and “Depressed or sad mood” built the strongest correlation pathway to connect anxiety and depression. Sleep problems and anxiety were proven to deteriorate interactively (60, 61). Neuroimaging studies suggest that total sleep loss amplifies activities in brain regions related to anxiety, such as the limbic system (62). Molecular imaging posits that neurotransmitter mechanisms underlying sleep-wake regulation are involved in anxiety (63). In addition, psychomotor agitation is a potentially violent syndrome characterized by uncontrollable motor increases and psychological and emotional activities (64, 65); as a marker of depression severity, psychomotor retardation is characterized by persistent slowness of cognitive and motor processing in speech, thinking and movements (66, 67). In fact, psychomotor retardation may be more common in functionally impaired elderly individuals, and the inhibition and poverty of thoughts caused by psychomotor retardation may relieve the nervousness of functionally impaired elderly individuals.

A4 “Trouble relaxing” was positively correlated with D7 “Concentration difficulties” and negatively correlated with D5 “Appetite changes.” Concentration is the ability to use cognitive resources to selectively inhibit the interference of irrelevant stimuli (68). Individuals with difficulties relaxing tend to consume more cognitive resources, which may lead to difficulty in concentration maintenance. Moreover, the functionally impaired elderly may improve their appetite if difficulties in relaxing is relieved.

A6 “Irritable” was positively correlated with D5 “Appetite changes” and D7 “Concentration difficulties.” Irritability represents an increased tendency to anger relative to peers (69), and a change in food intake can be a way to vent anger. Irritability is frequently comorbid with attention-deficit/hyperactivity disorder (70), and the impairments and dysfunction in individuals with irritability may be partially caused by concentration difficulties (71).

A7 “Afraid something will happen” was positively correlated with D9 “Thoughts of death.” In the functionally impaired elderly, a large part of the fear of the unknown is death anxiety. Existential theories point out that death anxiety is an inevitable process that is experienced before entering the level of consciousness (72); it describes the functionally impaired elderly's fear of death and is characterized by threat and discomfort (73).

### The intervention targets of anxiety and depression

A high value of EI indicates that a given symptom has a strong positive correlation with other symptoms in the network (74). Therefore, EI may play an important role in identifying potential intervention targets. In the present study, A2 “Uncontrollable worry” had the highest EI values in the network. Previous studies have found that “fatigue” has the highest centrality value in the network of nursing students, family workers and patients with major depressive disorder (40, 46, 75). This finding, which is inconsistent with previous studies, reflects the uniqueness of the anxiety and depression symptoms of functionally impaired elderly individuals. According to the cognitive avoidance theory of worry, imagery is prone to be more emotionally evocative than verbal-based thoughts; thus, for functionally impaired elderly people, the transition from threatening imagery to verbal-based worry can inhibit negative physiological arousal, and as a coping strategy, uncontrollable worry is negatively reinforced (76, 77). This negative coping strategy may lead to the interactive deterioration of anxiety and depression symptoms in functionally impaired elderly individuals. In addition, D2 “depressed or sad mood” also has a highest EI value and may provide a psychological intervention target for decreasing the severity of depression and anxiety. Depressed mood was found in 20% of free-living old people (78), and the proportion may be higher in the functionally impaired elderly. Moreover, depressed mood may be a predictor of daily living disability (79).

According to Jones et al. (35), bridge symptoms increase the risk of interaction between different disorders. The identification of bridge symptoms helps to clarify the complexity of comorbidity and provide potential intervention targets for comorbid disorders (80). In the present study, A6 “Irritable” and D7 “Concentration difficulties” were identified as bridge symptoms. At least 50% of major depressive disorder patients show irritability during the depressive episode (81), and irritability predicts a greater severity of major depressive disorder (82). Shared risk factors, including genetic risk, common temperamental characteristics and negative parenting styles, are used to explain the association between irritability and depression (83, 84). Similarly, concentration difficulties are common in anxiety disorders (85), and previous experimental evidence has confirmed the detrimental influence of anxiety on concentration (86).

### Suicide prevention for the functionally impaired elderly

As shown in the flow network, D9 “Thoughts of death” was directly related to most of the anxiety and depression symptoms. As one of the diagnostic symptoms of major depression (47), “thoughts of death” contains a series of contents, ranging from a passive wish of death to active suicide plans. The close relationship of death thoughts with anxiety and depression has been confirmed in many studies (87–91), and the fine-grained associations of death thoughts with distinct anxiety and depression symptoms revealed by network analysis reflect the complexity of death thoughts (46). The strongest direct edge of D9 “Thoughts of death” was with D6 “Feeling of worthlessness.” This finding was similar to the research of Wei et al. (44), who observed that “thoughts of death” were most correlated with “feeling of worthlessness” in the network of patients with epilepsy. However, according to Garabiles et al. (75) and Ren et al. (46), the strongest edge of “thoughts of death” was with “psychomotor agitation/retardation” in domestic workers and nursing students. This difference may be related to the health status of the participants; the need for long-term care due to illness or weakness leads to a feeling of worthlessness, and the feeling of worthlessness has been suggested as an indicator of an increased risk of suicide (92).

Although previous studies revealed that “thoughts of death” ranked the lowest centrality in the anxiety-depression network (33, 44), the centrality index of D9 “Thoughts of death” was not the lowest in the present study, which indicates the importance of death thoughts in the anxiety-depression network of the functionally impaired elderly. This result emphasizes the necessity of suicide prevention for functionally impaired elderly individuals. In addition to “thoughts of death,” “feeling of worthlessness” can be used as a potential target for suicide prevention.

### Limitations

Several limitations should be noted in this study. First, our research was based on cross-sectional data, and consequently, the temporal causality of anxiety and depression cannot be ascertained. Second, self-assessment scales were used in our study, and the results were susceptible to socially desirable responses. Third, although the difference tests of edge weight, centrality and bridge centrality have been widely used in network analysis (93–95), according to Epskamp et al. (32), the methods commonly used in correction for multiple comparison at present are not feasible in network analysis, and new method development is still a topic of future research. Fourth, considering the comorbidity of cognitive impairment, anxiety and depression among the functionally impaired elderly, network analysis including these three may be the direction of future research. Finally, the identification of intervention targets was based on network analysis theory; thus, testing these targets in practice is needed.

## Conclusion

In summary, the present study represents the first application of network analysis to comprehend the comorbidity of anxiety and depression in functionally impaired elderly individuals. The network structure helps to build a clear understanding of the fine-grained relationships between anxiety and depression in functionally impaired elderly individuals. The EI and BEI help to identify the central and bridge symptoms in the network and provide potential psychological intervention targets for efficiently reducing anxiety and depression. The flow network intuitively displays the relationship of death thoughts with other anxiety and depression symptoms and provides valid suggestions for suicide prevention. Our research enriches the theory of comorbidity of anxiety and depression and is of great significance to the practice of psychological intervention for functionally impaired elderly individuals.

## Data availability statement

The raw data supporting the conclusions of this article will be made available by the authors, without undue reservation.

## Ethics statement

The studies involving human participants were reviewed and approved by the Ethics Committee of Xijing Hospital, Air Force Medical University (No. KY20222194-C-1). The patients/participants provided their written informed consent to participate in this study.

## Author contributions

Concept and design: TY, SW, and XLiu. Acquisition of the data: XC, QZ, HW, and LW. Analysis and interpretation of the data: TY, XLi, and XW. Drafting of the manuscript: TY and ZG. Critical revision of the manuscript: XLiu, XZ, and SW. All authors contributed to the article and approved the submitted version.

## Funding

This study was funded by Air Force Medical University (BKJ19J021, BKJ21J013, and BWS16J012).

## Conflict of interest

The authors declare that the research was conducted in the absence of any commercial or financial relationships that could be construed as a potential conflict of interest.

## Publisher's note

All claims expressed in this article are solely those of the authors and do not necessarily represent those of their affiliated organizations, or those of the publisher, the editors and the reviewers. Any product that may be evaluated in this article, or claim that may be made by its manufacturer, is not guaranteed or endorsed by the publisher.

## Abbreviations

COVID-19: Coronavirus disease 2019
GAD-7: Generalized Anxiety Disorder-7
PHQ-9: Patient Health Questionnaire-9
DSM-IV: Diagnostic Manual Of Mental Disorders-IV
GGM: Gaussian graphical model
LASSO: Least absolute shrinkage and selection operator
EBIC: Extended Bayesian information criterion
EI: Expected influence
BEI: Bridge expected influence
CS-coefficient: Correlation stability coefficient.
