# Supplementary figures and images for: Network analysis of anxiety and depression in the functionally impaired elderly
Source: Front Public Health. 2022 Dec 1;10:1067646. doi: 10.3389/fpubh.2022.1067646 (PMC9751796; doi:10.3389/fpubh.2022.1067646)

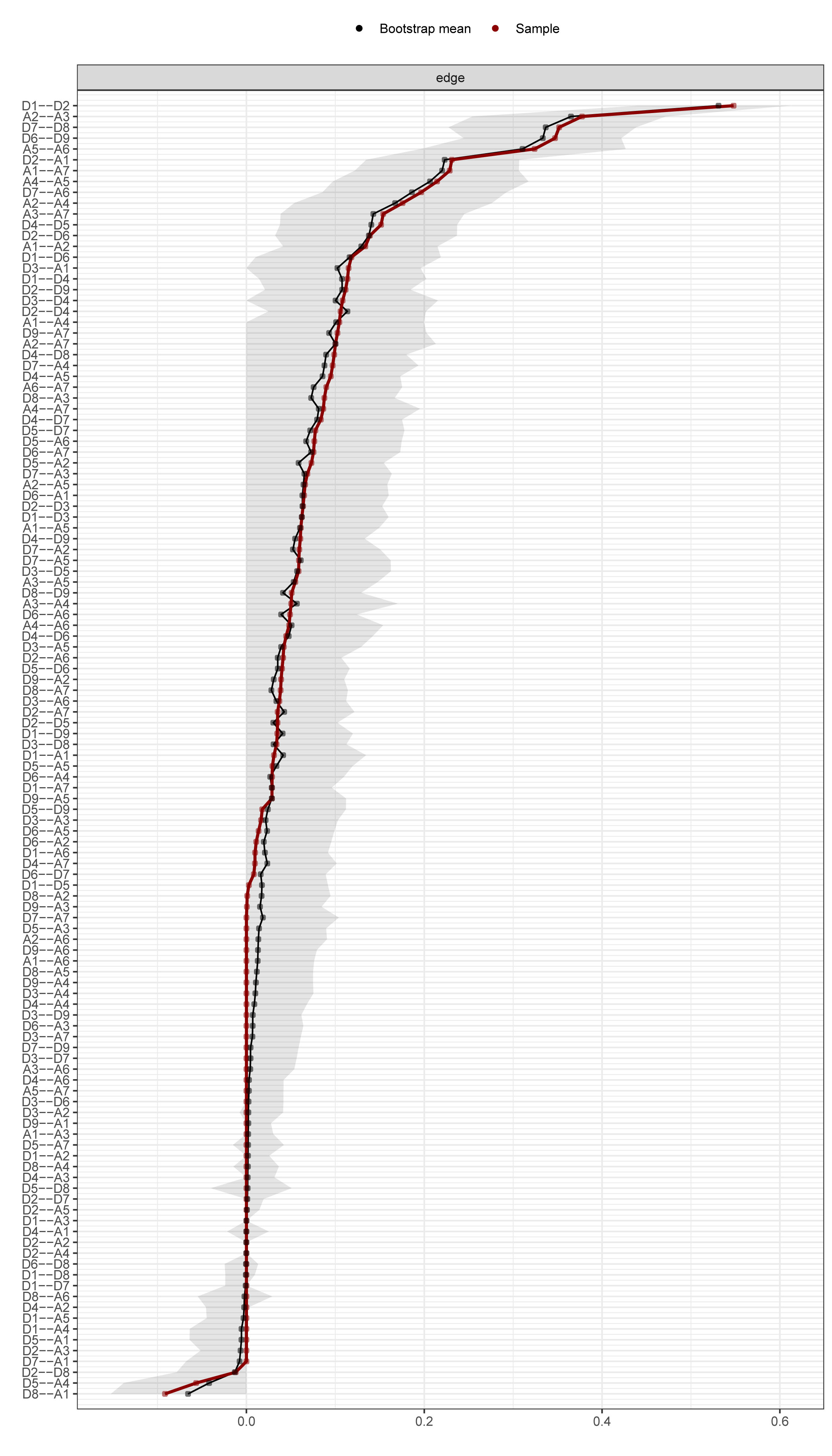

Supplement: Supplementary file 1 [file Image_1.JPEG]

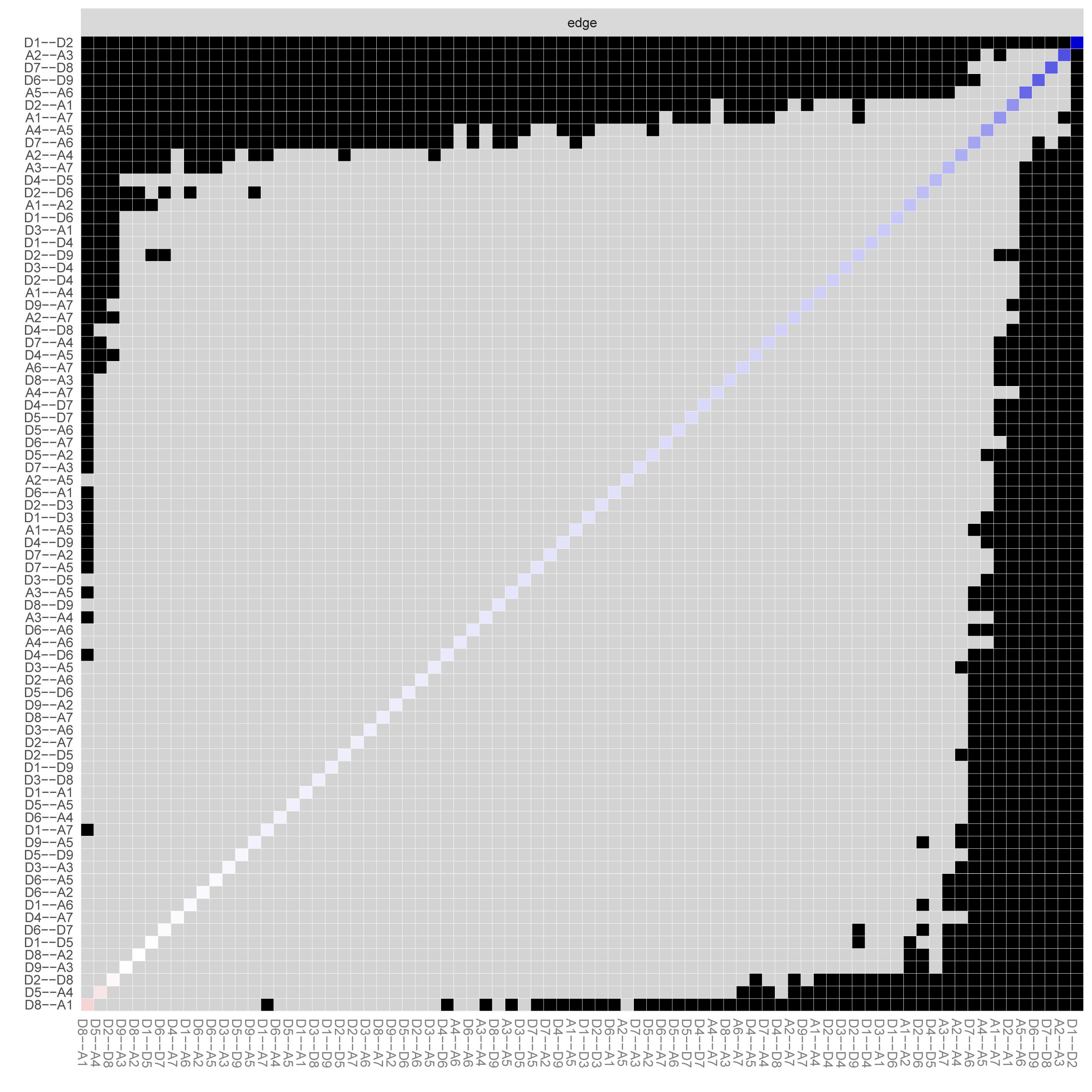

Supplement: Supplementary file 2 [file Image_2.JPEG]

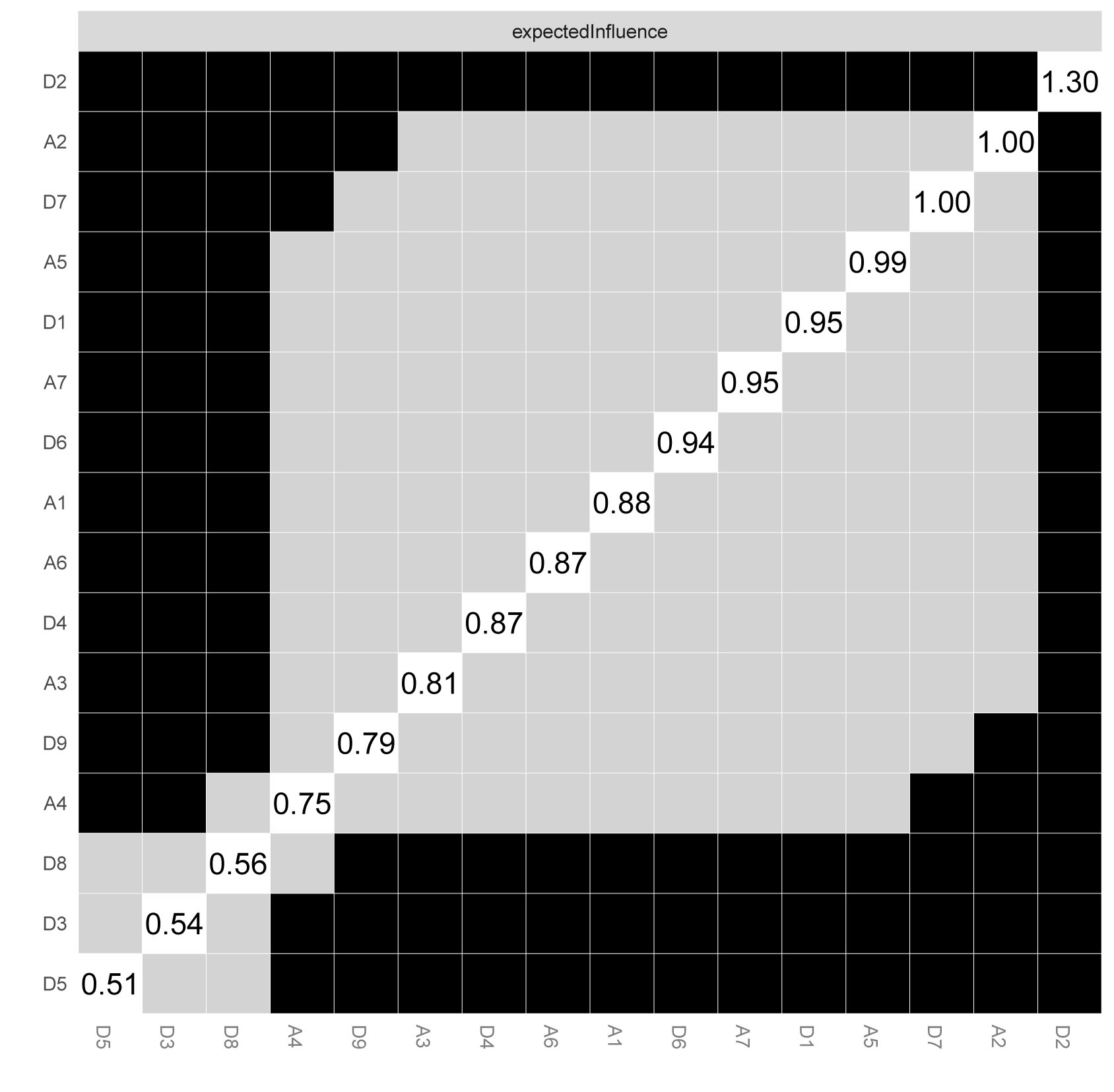

Supplement: Supplementary file 3 [file Image_3.JPEG]

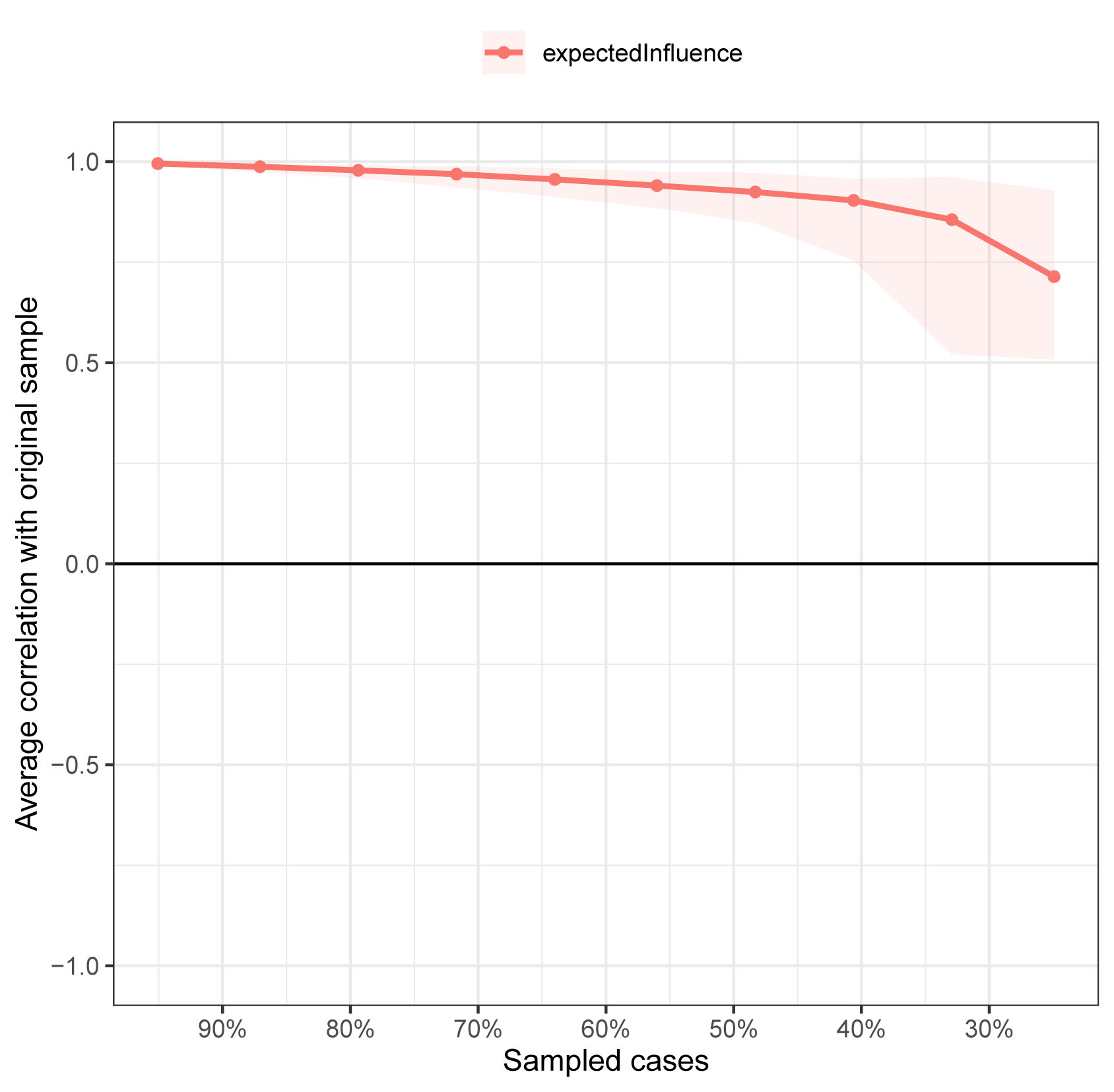

Supplement: Supplementary file 4 [file Image_4.JPEG]

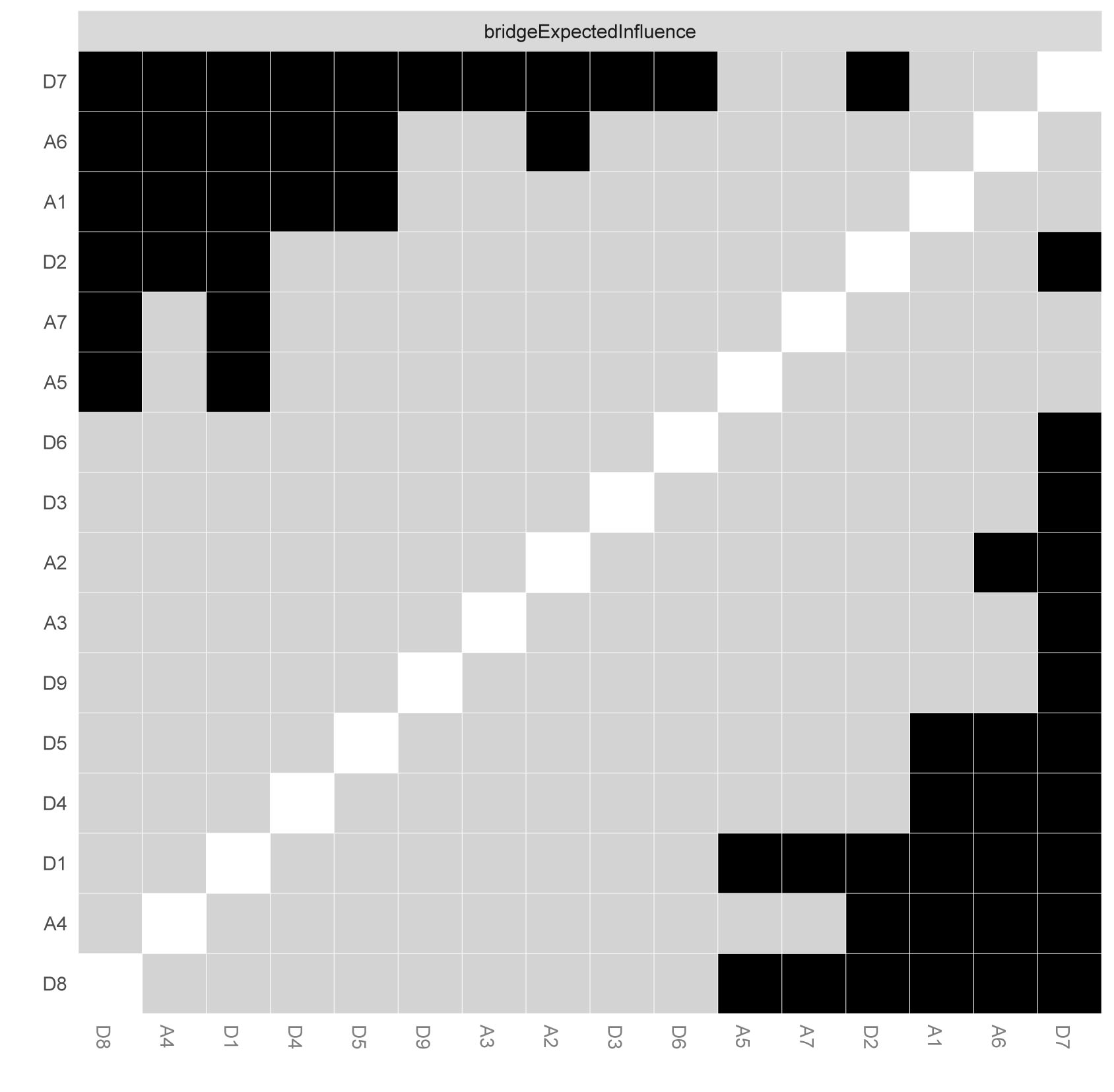

Supplement: Supplementary file 5 [file Image_5.JPEG]

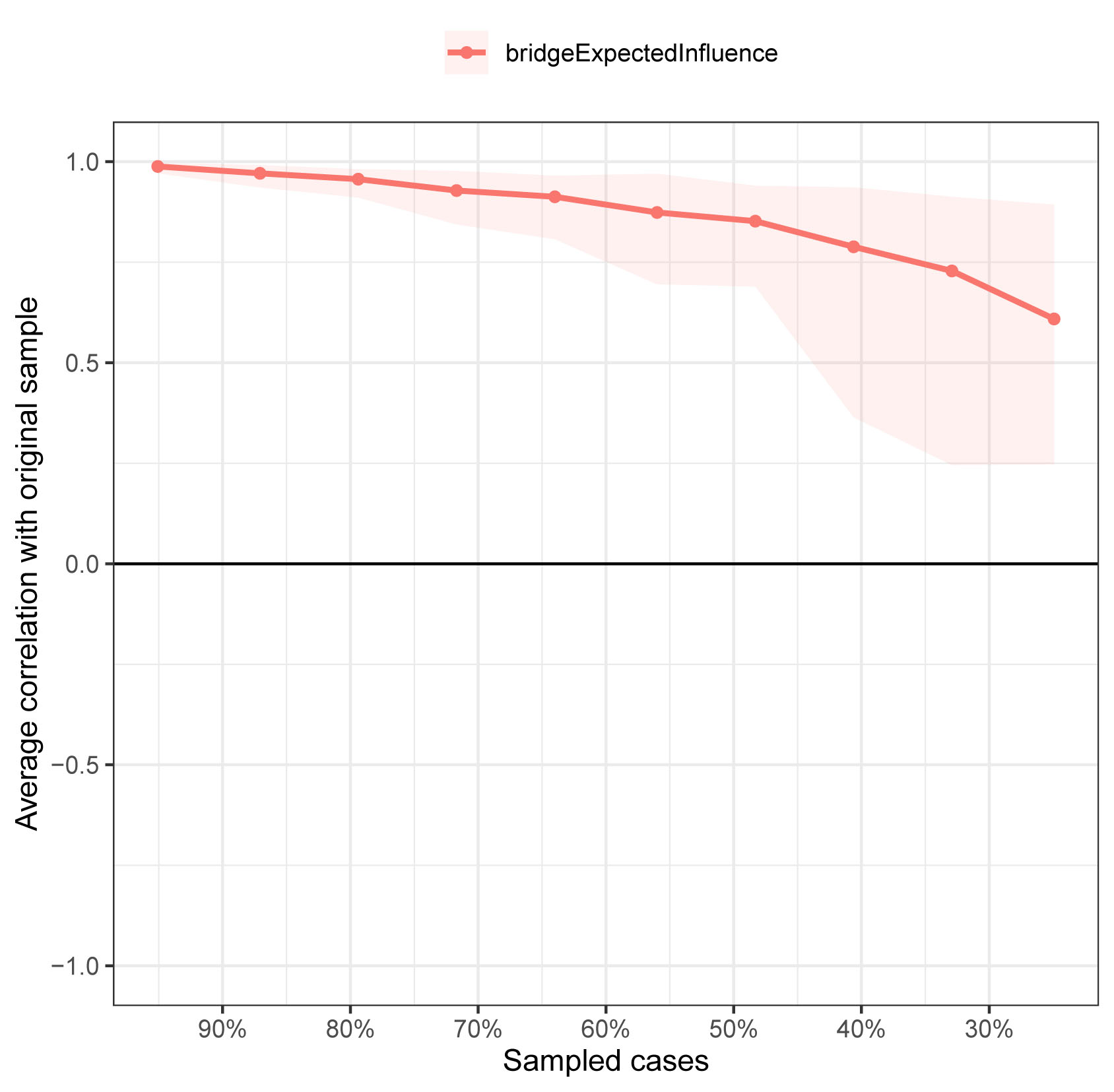

Supplement: Supplementary file 6 [file Image_6.JPEG]
