# Supplementary material for: Network analysis of anxiety and depression in the functionally impaired elderly
Source: Front Public Health. 2022 Dec 1;10:1067646. doi: 10.3389/fpubh.2022.1067646 (PMC9751796; doi:10.3389/fpubh.2022.1067646)
Supplement: Supplementary file 7 [file Table_1.DOCX]

Supplementary Table 1 The [Correlation](javascript:;) [Matrix](javascript:;) of the Anxiety-depression Network of the Functionally Impaired Elderly

|  | D1 | D2 | D3 | D4 | D5 | D6 | D7 | D8 | D9 | A1 | A2 | A3 | A4 | A5 | A6 | A7 |
| --- | --- | --- | --- | --- | --- | --- | --- | --- | --- | --- | --- | --- | --- | --- | --- | --- |
| D1 |  | 0.5479 | 0.0624 | 0.1137 | 0.0028 | 0.1181 | 0.0000 | 0.0000 | 0.0347 | 0.0310 | 0.0000 | 0.0000 | 0.0000 | 0.0000 | 0.0097 | 0.0288 |
| D2 | 0.5479 |  | 0.0637 | 0.1059 | 0.0350 | 0.1389 | 0.0000 | -0.0121 | 0.1115 | 0.2310 | 0.0000 | 0.0000 | 0.0000 | 0.0000 | 0.0413 | 0.0351 |
| D3 | 0.0624 | 0.0637 |  | 0.1082 | 0.0587 | 0.0000 | 0.0000 | 0.0339 | 0.0000 | 0.1149 | 0.0000 | 0.0165 | 0.0000 | 0.0420 | 0.0371 | 0.0000 |
| D4 | 0.1137 | 0.1059 | 0.1082 |  | 0.1515 | 0.0447 | 0.0837 | 0.0987 | 0.0607 | 0.0000 | 0.0000 | 0.0000 | 0.0000 | 0.0949 | 0.0000 | 0.0095 |
| D5 | 0.0028 | 0.0350 | 0.0587 | 0.1515 |  | 0.0401 | 0.0775 | 0.0000 | 0.0178 | 0.0000 | 0.0731 | 0.0000 | -0.0566 | 0.0293 | 0.0764 | 0.0000 |
| D6 | 0.1181 | 0.1389 | 0.0000 | 0.0447 | 0.0401 |  | 0.0083 | 0.0000 | 0.3471 | 0.0649 | 0.0110 | 0.0000 | 0.0289 | 0.0137 | 0.0490 | 0.0755 |
| D7 | 0.0000 | 0.0000 | 0.0000 | 0.0837 | 0.0775 | 0.0083 |  | 0.3517 | 0.0000 | 0.0000 | 0.0595 | 0.0686 | 0.0970 | 0.0591 | 0.1965 | 0.0000 |
| D8 | 0.0000 | -0.0121 | 0.0339 | 0.0987 | 0.0000 | 0.0000 | 0.3517 |  | 0.0509 | -0.0915 | 0.0009 | 0.0877 | 0.0000 | 0.0000 | 0.0000 | 0.0385 |
| D9 | 0.0347 | 0.1115 | 0.0000 | 0.0607 | 0.0178 | 0.3471 | 0.0000 | 0.0509 |  | 0.0000 | 0.0390 | 0.0005 | 0.0000 | 0.0287 | 0.0000 | 0.1024 |
| A1 | 0.0310 | 0.2310 | 0.1149 | 0.0000 | 0.0000 | 0.0649 | 0.0000 | -0.0915 | 0.0000 |  | 0.1337 | 0.0000 | 0.1045 | 0.0612 | 0.0000 | 0.2286 |
| A2 | 0.0000 | 0.0000 | 0.0000 | 0.0000 | 0.0731 | 0.0110 | 0.0595 | 0.0009 | 0.0390 | 0.1337 |  | 0.3775 | 0.1756 | 0.0660 | 0.0000 | 0.1002 |
| A3 | 0.0000 | 0.0000 | 0.0165 | 0.0000 | 0.0000 | 0.0000 | 0.0686 | 0.0877 | 0.0005 | 0.0000 | 0.3775 |  | 0.0503 | 0.0550 | 0.0000 | 0.1539 |
| A4 | 0.0000 | 0.0000 | 0.0000 | 0.0000 | -0.0566 | 0.0289 | 0.0970 | 0.0000 | 0.0000 | 0.1045 | 0.1756 | 0.0503 |  | 0.2144 | 0.0480 | 0.0865 |
| A5 | 0.0000 | 0.0000 | 0.0420 | 0.0949 | 0.0293 | 0.0137 | 0.0591 | 0.0000 | 0.0287 | 0.0612 | 0.0660 | 0.0550 | 0.2144 |  | 0.3241 | 0.0000 |
| A6 | 0.0097 | 0.0413 | 0.0371 | 0.0000 | 0.0764 | 0.0490 | 0.1965 | 0.0000 | 0.0000 | 0.0000 | 0.0000 | 0.0000 | 0.0480 | 0.3241 |  | 0.0899 |
| A7 | 0.0288 | 0.0351 | 0.0000 | 0.0095 | 0.0000 | 0.0755 | 0.0000 | 0.0385 | 0.1024 | 0.2286 | 0.1002 | 0.1539 | 0.0865 | 0.0000 | 0.0899 |  |
